# Anomalous Origin of Coronary Arteries Arising from the Right Coronary Cusp: A Rare Presentation

**DOI:** 10.7759/cureus.2535

**Published:** 2018-04-25

**Authors:** Rizwan Ali, Arooj Tahir, Mohammed I Shakhatreh, Kanna V Posina

**Affiliations:** 1 Internal Medicine, Rapides Regional Hospital, Alexandria, USA; 2 Cardiology, Rapides Regional Hospital, Alexandria, USA

**Keywords:** coronary anatomy, chest pain, anomalous coronary artery

## Abstract

The anomalous origin of coronaries is rare. The purpose of this case report is to show a rare anomalous origin of coronaries.

A 64-year-old female presented with chest pain that was typical in nature. The patient had left heart catheterization that showed an anomalous origin of coronaries, where all the coronaries were arising from the right coronary cusp. The patient had a significant disease in the distal left anterior descending artery, but it was a small vessel. Medical management was chosen.

This is a rare presentation of the coronary anatomy.

## Introduction

The anomalous origin of coronaries is rare [1]. According to the literature, coronary artery anomalies (CAAs) affect around 1% of the general population, ranging from 0.3%-5.6% in studies on patients undergoing coronary angiography and in approximately 1% of routine autopsies. The most common CAA is the separate origin of the left anterior descending (LAD) artery and the left circumflex (LCX) artery, with an incidence of 0.41%, followed by LCX arising from the right coronary artery (RCA), with an incidence of 0.37% [2-6].

## Case presentation

A 64-year-old African American female with a past medical history of insulin-dependent diabetes mellitus, hypertension, hyperlipidemia, prior history of stroke, hypothyroidism, and family history of coronary artery disease presented to the emergency department with complaints of typical chest pain. The patient's chest pain was associated with nausea and vomiting. The physical examination and initial electrocardiogram were unremarkable. Cardiac enzymes were negative. The patient was started on aspirin, statin, and nitroglycerin. Cardiology was consulted and they decided to do left heart catheterization through right radial access and an echocardiogram, as the patient was having unstable angina. The echocardiogram showed a normal ejection fraction with no wall motion abnormalities.

**Figure 1:**
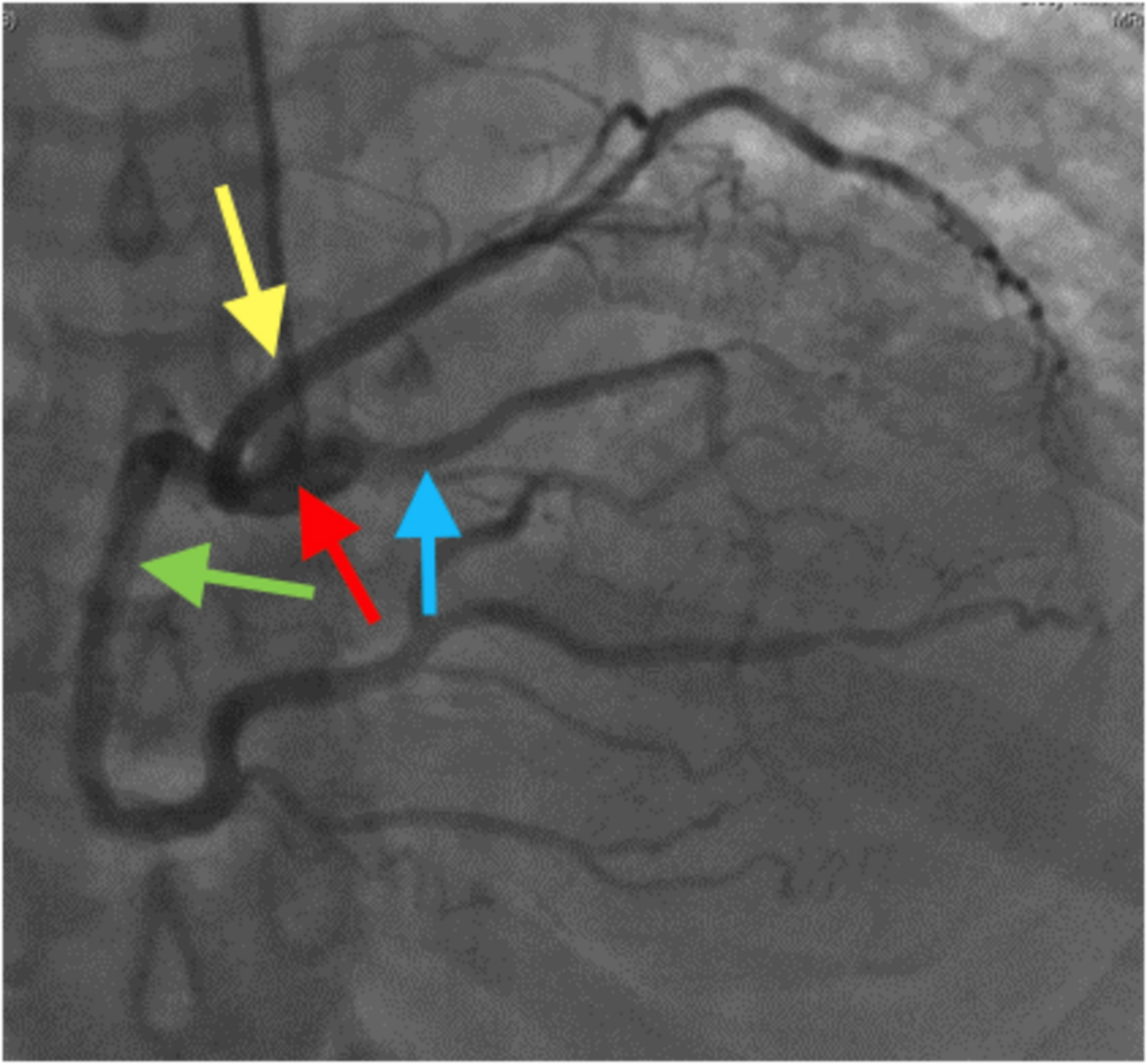
Left heart catheterization showing anomalous coronaries, with all three coronaries arising from the right coronary cusp with separate ostia. Yellow arrow: left circumflex artery; green arrow: right coronary artery; blue arrow: left anterior descending artery; red arrow: right coronary cusp

**Figure 2:**
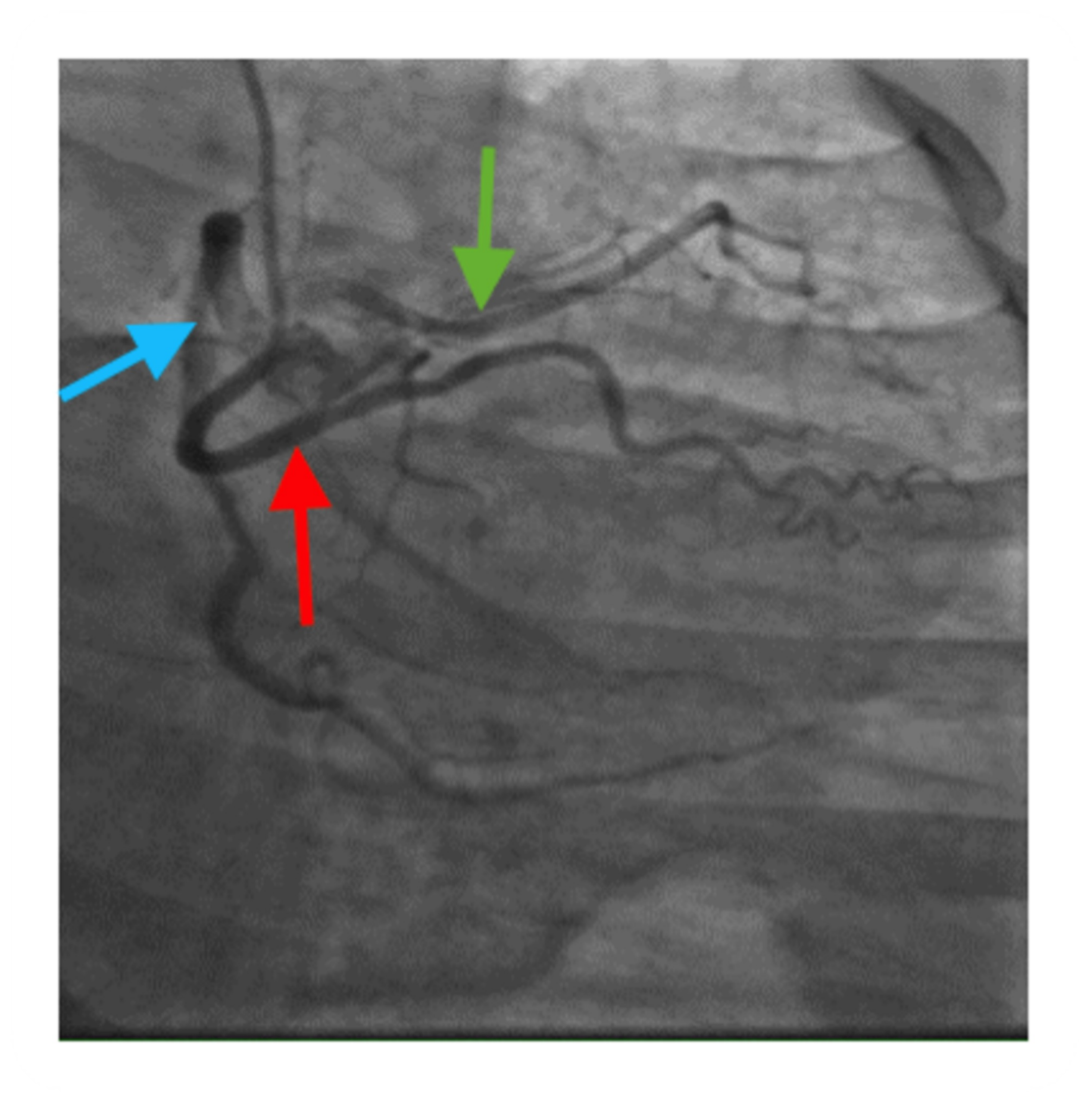
Another view of anomalous coronaries. Distal left anterior descending has a significant disease. Red arrow: left circumflex artery; blue arrow: right coronary artery; green arrow: left anterior descending artery

Left heart catheterization showed anomalous coronaries, with all three coronaries arising from the right coronary cusp with a separate ostium, as shown in Figures 1-2.

The left anterior descending artery (LAD) had an anomalous origin with a separate ostium from the right coronary cusp. There was focal moderate to severe 70%-80% disease in the mid vessel. The LAD was a small vessel. The left circumflex artery had an anomalous origin with a separate ostium from the right coronary cusp. Mild luminal irregularities were present. The right coronary artery was a large dominant vessel with mild luminal irregularities. It was decided to treat the patient with medical management.

## Discussion

This was a very rare presentation of a coronary anatomy. However, our patient did not have any problem with the anomalous origin of her coronaries. We think this type of anomaly is benign. However, the origin of the coronary artery arising from the innominate artery can be the cause of syncope [7] or chest pain in adults [8-9]. The origin of the right coronary artery from the descending thoracic aorta may be associated with atypical and striking elastotic changes and the thickening of the wall of the coronary artery as the underlying pathogenesis of severe consequences [10]. Circulatory symptoms may also be derived from the ectopic coronary arterial course between the pulmonary trunk and the aorta in spite of the lack of atherosclerotic plaques in the coronary artery [11]. The anomalous origin of the coronary artery can be associated with a common congenital heart defect [12] or with rare congenital heart defect like the cervical aortic arch [13]. The anomalous origin of the coronary artery can sometimes be associated with acquired heart disease, including coronary artery disease or heart valve disorders. Sudden death [14-15] and exercise-related death [16] are most common with the anomalous origin of the left main from the right coronary sinus. The anomalous origin of the right coronary artery from the left coronary sinus is also frequently associated with exercise-related sudden death. The high-risk anatomies responsible for sudden death are a coronary artery segment coursing between the pulmonary artery and the aorta [17], an acute angle take-off of the left coronary artery [18], and ostial abnormalities, including an ostial valve-like ridge [19], a slit-like orifice, and a flute beak-shaped ostium. The management of the anomalous origin of the coronary artery remains controversial. Surgical treatment is a definitive therapy that is recommended even for asymptomatic high-risk patients.

## Conclusions

A non-dominant coronary artery disease can be managed with medical management. However, the origin of the coronary artery arising from the innominate artery can be the cause of different symptoms, even sudden death. The high-risk anatomies responsible for sudden death are a coronary artery segment coursing between the pulmonary artery and the aorta, an acute angle take-off of the left coronary artery, and ostial abnormalities, including an ostial valve-like ridge, a slit-like orifice, and a flute beak-shaped ostium. The management of the anomalous origin of the coronary artery remains controversial. Surgical treatment is a definitive therapy that is recommended even for asymptomatic high-risk patients.
